# Effects of Epigallocatechin Gallate on the Stability of Epicatechin in a Photolytic Process

**DOI:** 10.3390/molecules24040787

**Published:** 2019-02-22

**Authors:** Shiuh-Tsuen Huang, Yi-An Hung, Meei-Ju Yang, Iou-Zen Chen, Jeu-Ming P. Yuann, Ji-Yuan Liang

**Affiliations:** 1 Department of Science Education and Application, National Taichung University of Education, Taichung 40306, Taiwan; hstsuen@mail.ntcu.edu.tw; 2 Department of Biotechnology, Ming-Chuan University, Gui-Shan 33343, Taiwan; amyhung840809@gmail.com; 3 Tea Research and Extension Station, Taoyuan 32654, Taiwan; 762204@gmail.com; 4 Department of Horticulture and Landscape Architecture, National Taiwan University, Taipei 10617, Taiwan; chenyo@ntu.edu.tw

**Keywords:** blue light, catechin, epicatechin, EGCG, gallic acid

## Abstract

Catechins belonging to polyhydroxylated polyphenols are the primary compounds found in green tea. They are associated with many physiological properties. Epicatechin (EC) is a non-gallate-type catechin with four phenolic hydroxyl groups attached. The changes in EC treated with color light illumination in an alkaline condition were investigated by chromatographic and mass analyses in this study. In particular, the superoxide anion radical (O_2_•^−^) was investigated during the EC photolytic process. EC is unstable under blue light illumination in an alkaline solution. When EC was treated with blue light illumination in an alkaline solution, O_2_•^−^ was found to occur via a photosensitive redox reaction. In addition, the generation of monomeric, dimeric, and trimeric compounds is investigated. On the other hand, epigallocatechin gallate (EGCG), which is a gallate-type catechin, is stable under blue light illumination in an alkaline solution. Adding EGCG, during the blue light illumination treatment of EC decreased photolytic formation, suggesting that gallate-type catechins can suppress the photosensitive oxidation of EC. Gallate-type catechins are formed via the esterification of non-gallate-type catechins and gallic acid (GA). The carbonyl group on the gallate moiety of gallate-type catechins appears to exhibit its effect on the stability against the photosensitive oxidation caused by blue light illumination.

## 1. Introduction

Polyphenols are polyhydroxylated compounds and secondary metabolites in plant products. These phenolic compounds have one or more hydroxyl groups attached to an aromatic ring and the structure may consist of a simple phenolic compound to a complex polymer [1]. Polyphenols also have beneficial characteristics, including anti-oxidative activity, as well as anti-radiation and anti-microbial activities [2].

**Figure 1:**
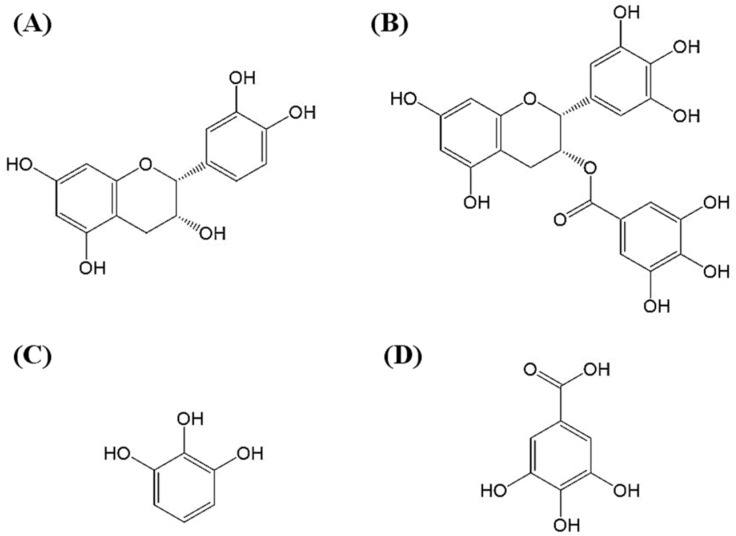
The molecular structures of (**A**) epicatechin (EC), (**B**) epigallocatechin gallate (EGCG), (**C**) pyrogallol (PG), and (**D**) gallic acid (GA).

Catechins are a class of polyphenolic compounds that are most commonly found as a natural product in foods and beverages, such as cacao, cacao products, grapes, red wine, green tea, and so forth. According to the gallic acid (GA) ester at the C_3_ position of the catechins, two major catechin types comprise about 80% of the polyphenolic compounds found in green tea: non-gallate-type catechins, such as epicatechin (EC) and catechin; gallate-type catechins, such as epicatechin gallate (ECG) and epigallocatechin gallate (EGCG) [3]. The structures of EC, EGCG, pyrogallol (PG), and GA are shown in Figure 1.

One of the significant disadvantages of catechins is their instability [4]. Catechins are easily oxidized, owing to the loss of hydrogen atoms in solution, with the generation of quinone-oxidized products and semiquinone radical species occurring via oxidation processes [5,6]. Catechin and EC are isomers that are structurally similar. It has been reported that catechin is stable in acidic conditions, but the color of catechin solution at pH 7.4 was altered after 96 h incubation [7]. Catechin is not stable in neutral and alkaline conditions. The catechin epimerization (i.e., the reaction with bicarboxylic acid via cross-linkage) can be achieved in neutral or alkaline conditions under hydrothermal treatment [2,8].

Catechins are unstable under UV light illumination. It was previously reported that EC and catechin were ultraviolet B (UVB)-sensitive and that yellow photoproducts were produced by the treatment with UVB radiation, whereas other catechins (ECG and EGCG) were relatively UVB-insensitive [9]. The photolysis of EC and catechin cleaves the ether bond of the flavan-3-ols heterocyclic ring and the epimerization of catechin is achieved via UV light illumination [10,11]. Catechin is also unstable under visible light illumination. Catechin is sensitive under blue light illumination in an alkaline solution with the superoxide anion radical (O_2_•^−^), and a chromogenic catechin dimer being generated during catechin photolysis via an electron-transfer mechanism [12,13]. EGCG is a gallate-type catechin compound and the most abundant one among tea catechins [14]. It is uncertain whether the peroxidation response exists or whether the free radicals are produced during the photolysis of the two major types of catechins treated with blue light illumination.

Catechin is unstable under blue light illumination and the catechin dimer can be suppressed in the presence of ascorbic acid during catechin photolysis in an alkaline solution, suggesting that ascorbic acid can inhibit catechin oxidation caused by photolysis [13]. Acting as antioxidants that scavenge diffusible free radical species, tea polyphenols are considered one of the main healthy components of tea beverages. It would be of interest to study how anti-oxidative reagents, such as gallate-type catechins (EGCG), decrease the photolytic oxidation of non-gallate-type catechins (EC) under blue light illumination in an alkaline solution.

The structural difference between epigallocatechin (EGC) and EC is an additional hydroxyl group in EGC at the 5′-position of the B ring. EGCG is formed via the esterification of EGC, comprising an ester derivative at the 3′-hydroxyl position on the C ring with a moiety gallate [15]. It would be of interest to find out whether attaching to a gallate group, by esterification, can enhance the stability of EGCG under blue light illumination in an alkaline solution.

Reactive oxygen species (ROS) such as hydrogen peroxide (H_2_O_2_), the hydroxyl radical (•OH), the superoxide anion radical (O_2_•^−^), and the peroxyl radical (ROO•), are reactive in general [16]. The O_2_•^−^, mostly formed as an intermediate, occurs via the reduction of oxygen, leading to harm to the organized system, inflammation, and atherosclerosis in addition to aging cells [17,18]. Previously, Liang et al. reported that the ROS from blue light-excited riboflavin or riboflavin-5′-phosphate (FMN) can cleave the plasmid DNA in integrity assays and inactivate *Escherichia coli* or methicillin-resistant *Staphylococcus aureus* by ROS formation [19,20,21]. It has been reported that O_2_•^−^ can be generated from catechin hydrate via blue light photolysis and can cause the inactivation of *Acinetobacter baumannii* (*A. baumannii*) [13]. EC is a non-gallate-type catechin with four phenolic hydroxyl groups. Further research will be aimed at investigating the ROS generated from EC photolysis.

Catechins are the largest fraction of polyphenols in green tea. The photolysis of two types of catechins (non-gallate- and gallate-type catechins) treated with blue light illumination was investigated in this study. This study was directed towards understanding the effects of blue light illumination on the changes in EC and the influence that EGCG or GA exerts on EC in a photoreaction system by chromatography and mass spectrometry techniques. The production of O_2_•^−^ from light-excited EC and the structural changes in EC treated with blue light were also examined.

## 2. Results

### 2.1. Effects of Blue, Green, and Red Lights on EC Photolysis

**Figure 2:**
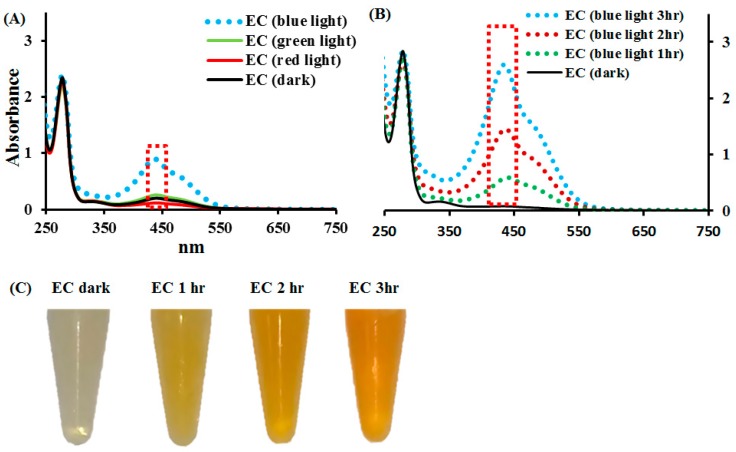
The absorption spectra of (**A**) 1 mM EC in 0.1 M phosphate buffer solution (ECPB) at pH 8, illuminated by blue, green, and red lights at 2.0 mW/cm^2^ for 1 h, (**B**) ECPB treated with or without blue light illumination at 2.0 mW/cm^2^ for 1, 2, and 3 h, and (**C**) the color changes in ECPB treated with or without blue light illumination at 2.0 mW/cm^2^ for 1, 2, and 3 h. The absorbance of the reaction solutions was measured within the spectral range from 250 to 750 nm.

Firstly, 1 mM EC in 0.1 M phosphate buffer solution (ECPB), treated with color light illumination, was examined. Figure 2A shows the spectra (250–750 nm) of ECPB at pH 8.0 metered by blue, green, and red light illumination. As observed, there was one absorption peak at 275 nm for EC in the dark. The absorbance of EC at 438 nm was significantly increased by blue light illumination, showing the highest efficiency in EC photolysis. The green and red lights exhibited little influence as the changes in the spectra at 438 nm were of no significance. Figure 2B,C show the effects of the illumination time of blue light on EC photolysis. EC, when in an alkaline solution, turned yellow under blue light illumination. As observed, the photochemical reaction of EC increased with the illumination time.

### 2.2. Detection of O_2_•^−^ in EC Photolysis

**Figure 3:**
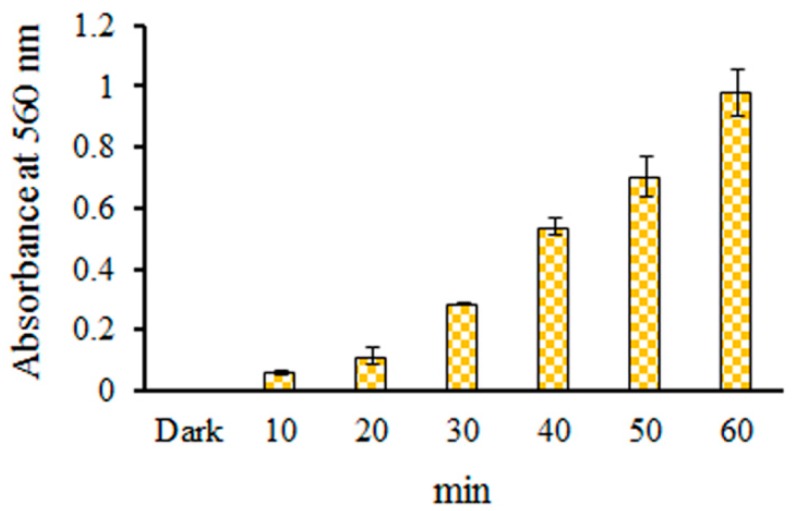
Effects of 1 mM EC on nitro blue tetrazolium (NBT) reduction by blue light irradiation at 2.0 mW/cm^2^ for 10–60 min. The results are represented by mean ± standard deviation, where *n* = 3. Statistically significant differences (*p* < 0.05) between each treatment are indicated by the different letters above each bar.

The production of O_2_•^−^ from catechin hydrate or tetracycline under blue light illumination was investigated using the nitro blue tetrazolium (NBT) reduction method [13,22]. The reductions in the EC/NBT system upon blue light illumination at 2.0 mW/cm^2^ for 10, 20, 30, 40, 50, and 60 min are shown in Figure 3. Figure 3 reveals that the photochemical effect of NBT reduction in EC was found to increase in a time-dependent manner under blue treatment. O_2_•^−^ was generated from EC under blue light illumination in phosphate buffer solution (PBS) at pH 7.8, suggesting that EC photolysis can be applied as a photosensitive oxidation process using generated O_2_•^−^ in the EC/NBT system.

### 2.3. Effects of Blue Light on EC, EGCG, GA, and PG Photolysis

**Figure 4:**
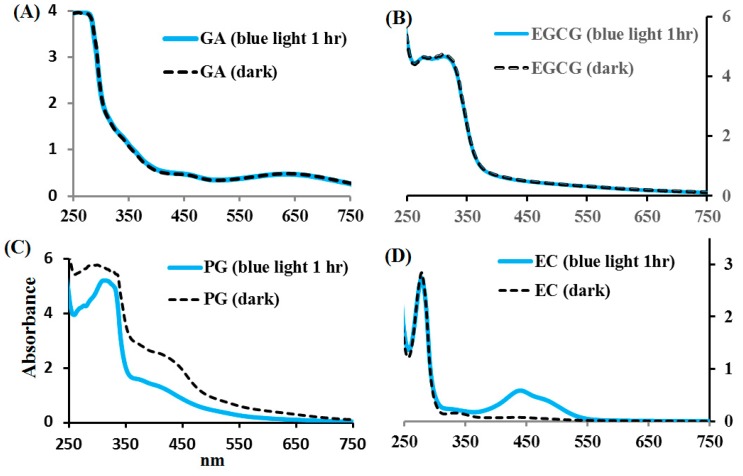
The absorption spectra of (**A**) GA, (**B**) EGCG, (**C**) PG, and (**D**) EC in phosphate buffer solution at pH 8.0, illuminated by blue light at 2.0 mW/cm^2^ for 1 h. The absorbance of the reaction solutions was measured within the spectral range from 250 to 750 nm.

Unlike EC, which is a non-gallate-type catechin, EGCG is a gallate-type catechin. PG is a trihydroxybenzene, while GA is a trihydroxybenzoic acid. The effects of blue light on EC, EGCG, GA, and PG photolysis were examined herein. The spectra of GA and EGCG in PBS at pH 8.0, treated with or without blue light illumination at 2.0 mW/cm^2^ for 1 h, were unchanged, as shown in Figure 4A,B. However, when EC and PG in PBS were treated with blue light illumination at 2.0 mW/cm^2^ for 1 h, the spectra were significantly altered, as shown in Figure 4C,D.

### 2.4. Effects of EGCG and GA on the EC Photolysis

**Figure 5:**
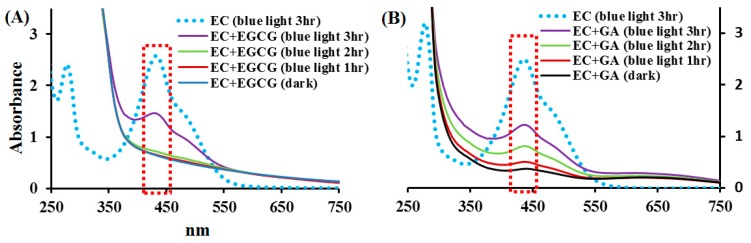
The absorption spectra of (**A**) ECPB at pH 8.0 treated with EGCG with illumination by blue light at 2.0 mW/cm^2^ for 1, 2, and 3 h; and (**B**) ECPB treated with GA and illuminated by blue light at 2.0 mW/cm^2^ for 1, 2, and 3 h. The absorbance of the reaction solutions was measured within the spectral range from 250 to 750 nm.

The effects of EGCG and GA on the spectral changes in ECPB at pH 8.0 under blue light illumination were studied. The absorption spectra of 1 mM ECPB treated with the same concentration of EGCG or GA, under blue light illumination at 2.0 mW/cm^2^ for 1, 2, and 3 h, achieve different levels of photolysis, as shown in Figure 5. It is also observed that ECPB under blue light illumination shows two peaks at 275 and 438 nm, as shown in Figure 5A,B. However, when ECPB was treated with 1 mM EGCG, under blue light illumination at 2.0 mW/cm^2^ for 1 and 2 h, the absorbance at 438 nm was suppressed in Figure 5A. By quantifying the absorbance at 438 nm, the extent of the ECPB reduction percentage upon blue light illumination at 2.0 mW/cm^2^ for 3 h was 57.9% in the presence of EGCG, suggesting that the addition of EGCG can inhibit the photosensitized oxidation of EC under blue light illumination.

The effect of GA on ECPB photolysis was similar to that on EGCG, as shown in Figure 5B. For the absorbance at 438 nm, the extent of the ECPB reduction percentage upon blue light illumination at 2.0 mW/cm^2^ for 3 h was 64.5% for the GA treatment under the same condition. The photolytic process of EC under blue light illumination can be inhibited by EGCG and GA.

### 2.5. Molecular Identification by LC–MS/MS Analysis

**Figure 6:**
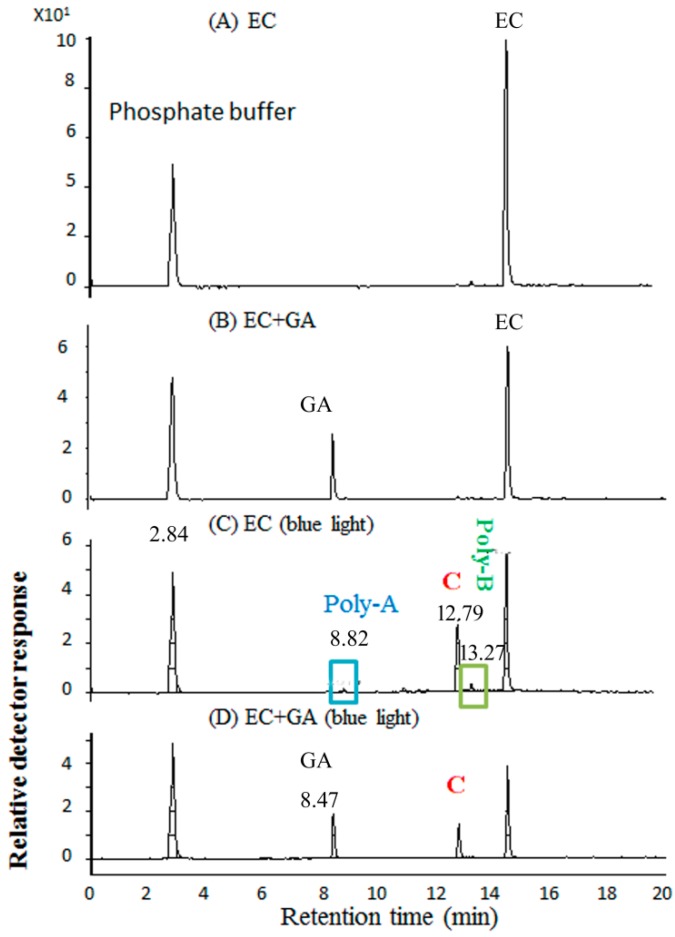
Total ion chromatogram of an HPLC–MS analysis of (**A**) ECPB at pH 8.0 in the dark, (**B**) ECPB treated with GA in the dark, (**C**,**D**) ECPB treated with blue light illumination at 2.0 mW/cm^2^ for 2 h in the absence and presence of GA, respectively.

EC solutions with basified and photochemical products were examined via an LC–MS/MS analysis. As shown in Figure 6, the total ion chromatogram of the PBS at pH 8.0 was observed at 2.84 min. In Figure 6A, the total ion chromatogram of the ECPB at pH 8.0 without photolysis processing was observed at 14.6 min (EC), and four signals were observed at 8.82 (Poly-A), 12.79 (C), 13.27 (Poly-B), and 14.6 min (EC) after blue light illumination in Figure 6C. As shown in Figure 2, for 1.0 mM ECPB at pH 8.0, treated with blue light illumination for 2 h, the absorption peak at 438 nm was significantly increased. An EC reduction percentage of 58.6% was observed under the same conditions by HPLC analyses in Figure 6. In Figure 6B, the total ion chromatogram of the ECPB treated with GA without photolysis processing was observed at 8.47 (GA) and 14.6 min (EC), whereas three signals could be observed at 8.47 (GA), 12.79 (C), and 14.6 min (EC) after blue light illumination, as shown in Figure 6D.

**Figure 7:**
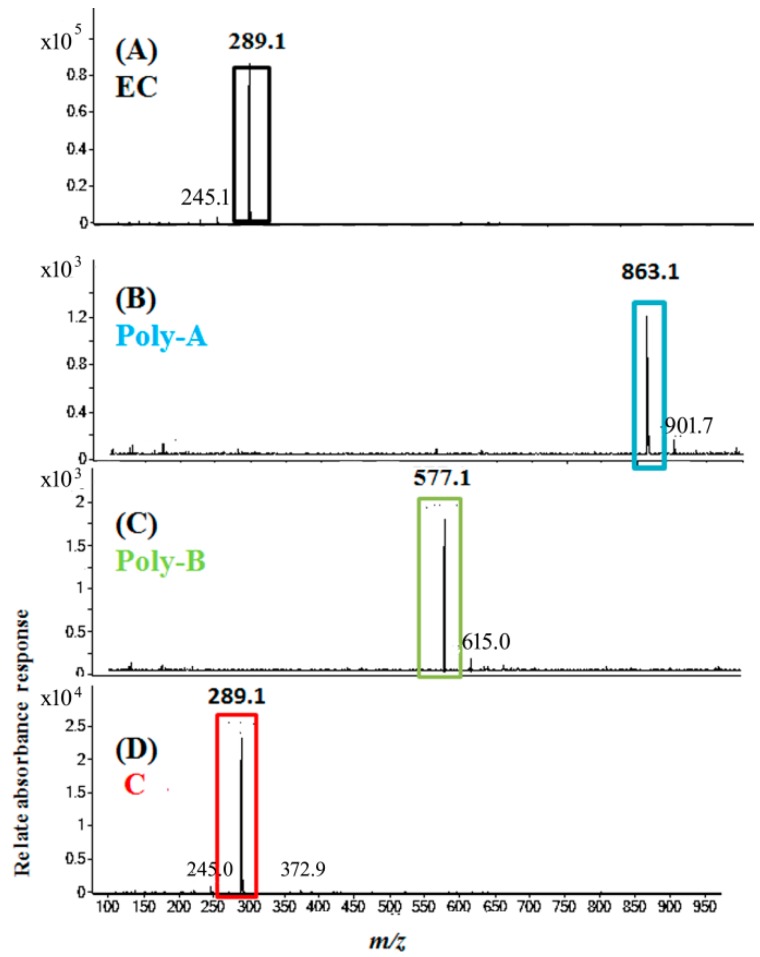
Electron ionization mass spectra of EC and the photoreaction products from ECPB, illuminated by blue light for 2 h. Product ion spectra of [M–H]^−^ for a blue-light-treated ECPB at pH 8.0. The precursor ion selected is m/z 289.1 and the proposed fragments are shown.

Figure 7 shows the electron ionization mass spectra after analyses from ECPB photolysis. As shown in Figure 7A, EC was observed at 14.6 min, with the major ion fragment being m/z 289.1. Poly-A and Poly-B were the photolysis products that were observed at 8.82 and 13.27 min, respectively, with their major ion fragments being m/z 863.1 and 577.1, respectively, as shown in Figure 7B,C. The emerging peak C was observed at 12.79 min, very similar to catechin eluted at the same retention time (data not shown). In addition, the compound of peak C, one of the EC photolytic products, was verified by its mass spectrum, with the major ion being m/z 289.1, in Figure 7D and thus identified as catechin. The signals were attributed to the quasi-molecular ions [M–H]^−^ in mass spectra. The molecular weights of Poly-A and Poly-B were supposedly 863.1 and 577.1 Da, respectively.

### 2.6. HPLC–DAD Analysis of EGCG Under Blue Light Illumination

**Figure 8:**
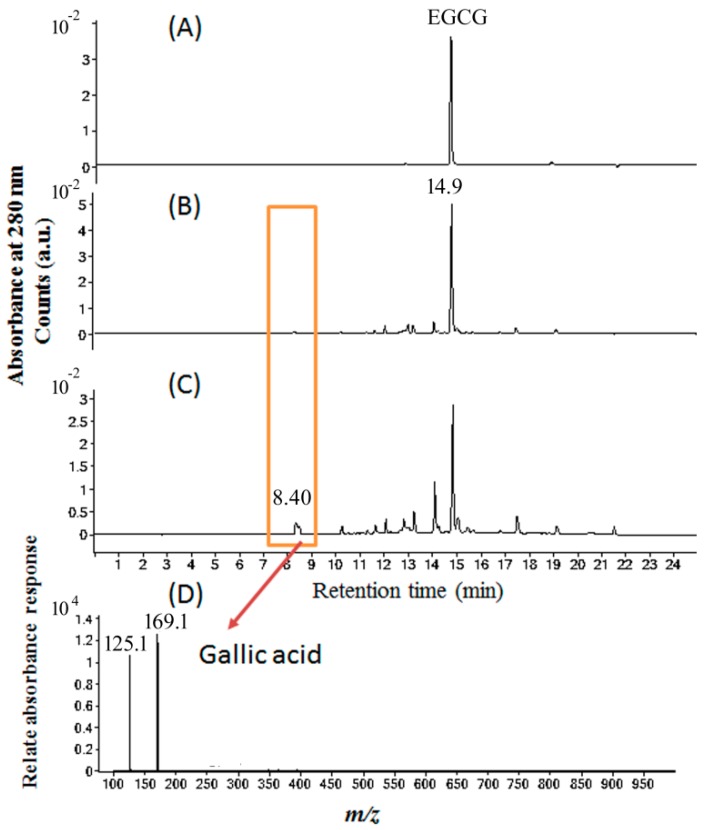
Chromatograms of HPLC–photodiode-array detector (HPLC–DAD) analyses of (**A**) EGCG in H_2_O, (**B**) EGCG in phosphate buffer solution (pH 8), (**C**) EGCG in phosphate buffer solution (pH 8) treated with blue light illumination at 2.0 mW/cm^2^ for 2 h, and (**D**) electrospray ionization mass spectra of the photoreaction products from the EGCG in (**C**) at 8.4 min.

As shown in Figure 8, the chromatograms of EGCG after blue light illumination were analyzed by an HPLC–photodiode-array detector (HPLC–DAD) at 280 nm. The pH of 1 mM EGCG in pure water was 6.2 and pH of EC was 6.9. EGCG dissolved in H_2_O was observed at 14.9 min and was determined to be stable, as shown in Figure 8A. For EGCG in PBS at pH 8, many other chromatographic signals were also found, as shown in Figure 8B. After blue light illumination, the photoreaction products of EGCG in PBS were observed in Figure 8C, with GA being confirmed at 8.40 min by its mass spectrum of m/z 169.1, as shown in Figure 8D.

## 3. Discussion

Yang et al. reported that the ROS from blue light-excited catechin hydrate can lead to the inactivation of *A. baumannii* by ROS formation [13]. In this study, EC at pH 7.8, under blue light illumination, generates O_2_•^−^ by aerobic photo-oxidative processes. The slope of the NBT reaction curve for the photolytic time (min) of EC can be applied as an index to show the production of ROS with respect to the standard. The slope of the NBT reaction curve of ROS production for EC was similar to that of catechin hydrate (0.018) photolysis under blue light illumination in the same conditions (data not shown). Under blue illumination, EC photolysis reactions can also inactivate *A. baumannii* (data not shown). Therefore, EC can be considered as a potential photosensitizer for microbial inactivation by EC photosensitized reactions.

The effects of EGCG on the photo-oxidation stability of EC photolysis were examined in this study. Green tea infusion turning brown is an important issue for the shelf life of tea drinks in the tea industry and was shown to be due to the oxidation of polyphenols [13]. Shi et al. reported that catechin or an EC solution under UVB illumination can generate several new photoproducts via photo-induced electron transfer [9]. Yang et al. reported that catechin hydrate at pH 8.0 under blue light illumination can generate a chromogenic catechin dimer (proanthocyanidin) via photosensitized oxidation and photo-induced electron transfer. However, addition of ascorbic acid during the photochemical process can suppress the photosensitive oxidation of catechin [13]. Upon the addition of EGCG or GA, the inhibition of a photosensitized oxidation process of EC under blue light illumination was observed, as evidenced by the disappearance of the absorbance of EC at 438 nm.

EC (a non-gallate-type catechin) is sensitive to blue light illumination, while EGCG (a gallate-type catechin) in PBS at pH 8 is insensitive to change by the spectrophotometric method. GA comprises a PG moiety attached to a carboxylic acid group. It is shown in this study that GA in PBS at pH 8.0, treated with or without blue light illumination, was insignificantly changed by the spectrophotometric method, but PG at pH 8.0 is sensitive to blue light illumination. GA and PG contain a benzene ring attached to three hydroxyl groups, both showing a strong UV absorption capacity due to the π electrons of the benzene ring. PG in basified solution is not stable during photo-oxidation caused by blue light illumination. The stability of GA can be at least partly explained by its carboxylic acid group, an electron-withdrawing group that makes the three hydroxyl groups less likely to undergo photo-oxidation as it occurs in PG. In this study, EC and PG at pH 8.0 are sensitive to blue light illumination. Belonging to gallate-type catechins, EGCG is formed via the esterification of EGC (containing a B ring with a moiety PG) with a moiety of gallate attached to the C ring by ester linkage. At pH 8.0 and under blue light illumination, EGCG can generate GA, as shown in this study. By making benzene rings less electron-rich, the carbonyl group on the gallate moiety of EGCG exhibits its effect on the spectrophotometric change caused by blue light illumination.

**Figure 9:**
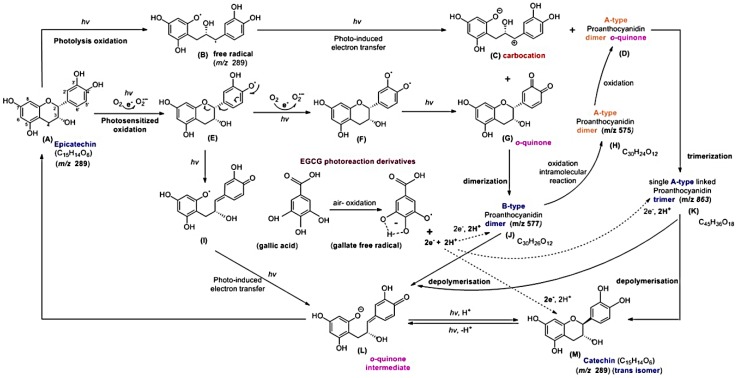
Proposed scheme for the mechanism of the EC photoreaction and the interactions of the intermediates with EGCG.

EC is stable in water and does not change the pH of its aqueous solution. However, in this study, EC at pH 8.0 was not stable under blue light illumination and the addition of EGCG or GA can inhibit the photosensitized oxidation process of EC. Moreover, monomeric, dimeric, and trimeric compounds were observed when ECPB was treated with photolysis processing at pH 8.0, but the product of ECPB treated with an anti-oxidative reagent (GA) under blue light illumination is only a monomeric one. Based on the results of this study, an EC photoreaction mechanism under the influence of EGCG and GA is proposed, as shown in Figure 9.

EC is a diastereomer of catechin and, unsurprisingly, they exhibit similar photochemical properties. For the photo-oxidation of flavan-3-ols, two plausible pathways are proposed (i.e., a single oxygen molecule reacts with the substrate), which results in the generation of radicals [13,23]. Additionally, by losing hydrogen atoms, the auto-oxidation of EC arises with the formation of quinone-oxidized products via semiquinone radical intermediates (Figure 9I), and O_2_•^−^ in the reaction mixture [5,6,13]. The irradiation of EC under blue light leads to the one-electron oxidation of the B ring, as shown by the appearance of O_2_•^−^. The –OH bond of the heterocyclic ring was excited via photosensitized oxidation, so as to form an *o*-quinone compound, as shown in Figure 9G. Owing to its low bond dissociation energy, the ether bond of the heterocyclic ring (ring C) of EC can be readily cleaved with the ring opened by photolysis, resulting in the generation of a free radical intermediate at m/z 289, as shown in Figure 9B [9,13]. The neutral radicals can also be ionized in a polar solvent via a photo-induced electron transfer reaction, with the product being a carbocation of EC, as shown in Figure 9C [13,24]. In Figure 9J, the formation of a B-type proanthocyanidin dimer at m/z 577 can be achieved from the condensation of the *o*-quinone compound and a carbocation intermediate of EC [13,25,26,27].

The constitutive building blocks of the B-type proanthocyanidin dimer, usually flavan-3-ols (e.g., (+)-catechin and (−)-epicatechin) can be linked together by a single bond through interflavan linkages [28]. It has been reported that an initial oxidative removal of hydride ion results in the oxidative transformation of B-type procyanidins into A-type procyanidins [29,30]. As shown in Figure 9H, an A-type procyanidin dimer derived from the B-type procyanidin dimer is observed at m/z 575. As shown in Figure 9D, further intramolecular oxidation causes the transformation of the A-type proanthocyanidin dimer into an A-type proanthocyanidin dimer *o*-quinone at m/z 573 [31]. The A-type proanthocyanidin trimer (m/z 863) can be generated from the condensation of the A-type proanthocyanidin dimer *o*-quinone and a carbocation intermediate of EC, as shown in Figure 9K.

In Figure 9L, the *o*-quinone intermediate was generated from semiquinone radical intermediates (Figure 9I) via a photo-induced electron transformation. The *o*-quinone intermediate, unstable in alkaline conditions, can be reduced to generate EC at m/z 289 (Figure 9A) or catechin at m/z 289 (Figure 9M) [13,32].

GA and EGCG are used as the antioxidants because of their strong reducing power. EGCG is also used for the treatment of superficial bladder cancer or to alleviate the symptoms of Down syndrome [33,34]. In this study, EGCG treated with blue light illumination at pH 8.0 can generate GA. GA is a trihydroxybenzoic acid and exhibits two pKa values, 4.1 and 8.38, for the carboxyl and hydroxyl groups, respectively [35]. It was reported that GA in an alkaline condition can be rapidly oxidized through the three hydroxyl groups attached to the aromatic ring that are prone to oxidation [36], resulting in a two-electron, two-proton, and anion gallate free radical oxidation scheme [37]. In Figure 9J,K, the disconnection within proanthocyanidins is achieved by the cleavage of C–C or C–O–C bonds between the B-type proanthocyanidin dimer and the A-type proanthocyanidin trimer, through the protons or quinone methide cleavage via GA in an alkaline condition [13,26,36,38,39]. Ultimately, a depolymerization process promotes the monomeric catechin (Figure 9M) and/or EC (Figure 9A) generation.

## 4. Materials and Methods

### 4.1. Chemicals

GA, EGCG, methanol, and PG were obtained from Sigma-Aldrich (St. Louis, MO, USA). NBT was obtained from Bio Basic, Inc. (Markham, ON, Canada). (−)-Epicatechin (EC) was purchased from Tokyo Chemical Industry Co. (Tokyo, Japan). Ultra-pure water by the Milli-Q system (Merck Millipore, Burlington, MA, USA) was used as a solvent throughout this study.

### 4.2. Illumination System

The illumination system comprises an opaque illumination chamber (height, 80 mm; diameter, 70 mm), and a power supply (YP30-3-2, Chinatech Co., New Taipei City, Taiwan) as described previously [13,22]. Six blue, green, or red LED lamps (DC 12 V 5050, vitaLED Technologies Co., Tainan, Taiwan) were applied to surround the inner walls of the chamber. The response solution was left within a glass test tube and maintained on the upper level of the chamber. The power supply and a solar power meter (TM-207, Tenmars Electronics Co., Taipei, Taiwan) were used to provide light illumination. The temperature of the photoreaction system was kept at 25 ± 3 °C. The wavelengths of the emitted maxima of the blue, green, and red lights were 463, 529, and 632 nm, with their W_1/2_ (spectral width at half height) being 23, 31, and 14 nm, respectively, as described previously [40].

### 4.3. Effects of Blue, Green, and Red Lights on EC Photolysis

The effects of blue, green, and red lights on EC photolysis were investigated by a UV/Vis spectrometer (Lambda35, Perkin-Elmer, Waltham, MA, USA). Briefly, (A) 1 mM EC in 0.1 M phosphate buffer solution (ECPB) at pH 8.0 in the dark was used as a standard control solution, (B) 1 mM ECPB was illuminated by blue, green, and red lights at 2.0 mW/cm^2^ for 1 h, and (C) 1 mM ECPB was illuminated by blue light at 2.0 mW/cm^2^ for 1, 2, and 3 h. The absorbance of the ECPB was recorded within the range of 250–750 nm by a UV/Vis spectrometer.

### 4.4. O_2_•^−^ Determination

A direct or an indirect method can be utilized for the detection of O_2_•^−^. Using the direct method requires a special apparatus, such as an electron paramagnetic resonance (EPR) spectrometer, whereas indirect assays are more broadly practiced in analyses [13]. The nitro blue tetrazolium (NBT) reduction method can be applied to detect O_2_•^−^ activity and is a widely used indirect method for the detection of O_2_•^−^ levels [40].

The effects of EC photolysis on O_2_•^−^ production were studied. The chemicals were freshly prepared prior to the NBT reduction method. The reactants comprised EC, methionine, and NBT of 1, 9, and 0.15 mM concentrations in 0.1 M phosphate buffer solution (PBS) at pH 7.8, respectively. The reactants were then treated with blue light illumination at 2.0 mW/cm^2^ for 10, 20, 30, 40, 50, and 60 min. The occurrence of O_2_•^−^ generated from EC photolysis was observed when NBT undergoes reduction to form blue formazan, which showed absorbance at 560 nm.

### 4.5. Effects of Blue Light on the Photolysis of EC, EGCG, GA, and PG

The effects of blue light on the photolysis of catechins studied herein were investigated via UV/Vis spectrophotometry. In brief, (A) 1 mM EC, EGCG, GA, or PG in 0.1 M PBS at pH 8.0 in the dark was used as a standard control solution, and (B) EC, EGCG, GA, or PG in 0.1 M PBS was illuminated by blue light at 2.0 mW/cm^2^ for 1 h. The absorbance of the reaction solutions was recorded within the range of 250–750 nm by a UV/Vis spectrometer.

### 4.6. Effects of EGCG or GA on EC Under Blue Light Illumination

The effects of EGCG or GA on EC under blue light illumination were examined spectrophotometrically. In brief, (A) 1 mM ECPB at pH 8.0 treated with either 1 mM EGCG or 1 mM GA in the dark was used as a standard control solution, (B) 1 mM ECPB was illuminated by blue light at 2.0 mW/cm^2^ for 1, 2, and 3 h, and (C) 1 mM ECPB was treated with 1 mM EGCG or 1 mM GA under blue light illumination at 2.0 mW/cm^2^ for 1, 2, and 3 h. The absorbance of the ECPB was recorded within the range of 250–750 nm by a UV/Vis spectrometer.

### 4.7. LC–MS/MS Analysis of EC Treated with GA Under Blue Light Illumination

ECPB and its related photolysis products subjected to an LC/MS/MS analysis were analyzed by an Agilent 6410B triple quadrupole LC/MS/MS and Agilent 1200 HPLC (Agilent Technologies, Palo Alto, CA, USA) connected with an electrospray ionization (ESI) source, as described previously [13]. The negative ion mode of the ESI technique was used in this study. Data were obtained by the Agilent Mass Hunter Workstation software version B.06.00.

The reaction solutions were eluted using an Agilent Poroshell 120 EC-C18 column (2.7µm, 4.6 × 150 mm, Agilent Technologies, Palo Alto, CA, USA). The effects of GA on EC under blue light illumination were examined via LC–MS/MS analysis. In brief, (A) 1 mM ECPB at pH 8.0 in the dark was used as a standard control solution, (B) 1 mM ECPB was illuminated by blue light at 2.0 mW/cm^2^ for 2 h, (C) 1 mM ECPB was treated with 1 mM GA in 0.1 M PBS at pH 8.0 in the dark, and (D) 1 mM ECPB was treated in the presence of 1 mM GA under blue light illumination at 2.0 mW/cm^2^ for 2 h.

The sample solution was filtered through a 0.45 μm filter (Millipore, Burlington, MA, USA) prior to its use. In the beginning, the mobile phase comprised 0.1% formic acid as solvent (A) and acetonitrile as solvent (B) with the gradient profile set as described previously [13]. The linear gradient started with 0–3 min, 1%–10% solvent B; 3–10 min, 10%–20% solvent B; 10–16.5 min, 20%–25% solvent B; and 16.6–20 min, 50%–100% solvent B; and returned to the original ratio at 23 min (i.e., 1% solvent B). Each sample of 10 μL was injected with a flow rate at 500 μL/min and the detection wavelength was set at 280 nm.

### 4.8. HPLC–DAD Analysis of EGCG Under Blue Light Illumination

EGCG and its relevant photoproducts were investigated in this study. In brief, (A) 1 mM EGCG in 0.1 M PBS at pH 8.0 in the dark was used as a standard control solution, and (B) 1 mM EGCG in 0.1 M PBS was illuminated by blue light at 2.0 mW/cm^2^ for 2 h. Both the HPLC–DAD and ESI mass spectral analyses of EGCG photolysis were examined as described in Section 4.7.

### 4.9. Statistics

The experiments were performed at least in triplicate. Data are expressed as the mean ± standard deviation of every single test. A one-way analysis of variance (ANOVA) was performed to identify the significant differences in more than three groups. When statistically significant differences were indicated, an unpaired Student’s t-test was used for further analysis. The difference of two groups of measurements was considered statistically significant when *p* < 0.05.

## 5. Conclusions

Chromatographic and mass analyses were applied to investigate the effects of photolysis on the conformational changes in epicatechin (EC) in an alkaline condition. EC is a non-gallate-type catechin and is sensitive to blue light illumination. Blue light illumination can; therefore, be efficient in terms of studying EC photosensitized oxidation. The epimerization of EC was achieved by epi-configuration with the dimeric and trimeric compounds being formed from the electron transfer of EC under blue light treatment in an alkaline condition. EGCG is a gallate-type catechin and is formed via the esterification of non-gallate-type catechins and GA. Addition of EGCG can inhibit the photosensitized redox reaction, such as ROS formation, and suppress the oxidation of EC caused by blue light illumination.
